# Structure–Function Relationships Underlying the Capacity of *Bordetella* Adenylate Cyclase Toxin to Disarm Host Phagocytes

**DOI:** 10.3390/toxins9100300

**Published:** 2017-09-24

**Authors:** Jakub Novak, Ondrej Cerny, Adriana Osickova, Irena Linhartova, Jiri Masin, Ladislav Bumba, Peter Sebo, Radim Osicka

**Affiliations:** 1 Institute of Microbiology of the CAS, v.v.i., 142 20 Prague, Czech Republic; jakub.novak@biomed.cas.cz (J.N.); o.cerny@imperial.ac.uk (O.C.); osickova@biomed.cas.cz (A.O.); linhart@biomed.cas.cz (I.L.); masin@biomed.cas.cz (J.M.); bumba@biomed.cas.cz (L.B.); sebo@biomed.cas.cz (P.S.); 2 Faculty of Science, Charles University in Prague, 128 43 Prague, Czech Republic

**Keywords:** adenylate cyclase toxin, *Bordetella*, β_2_ integrins, cAMP, CD11b/CD18, cell signaling, complement receptor 3, innate immunity, membrane pores, repeats-in-toxin

## Abstract

*Bordetellae*, pathogenic to mammals, produce an immunomodulatory adenylate cyclase toxin–hemolysin (CyaA, ACT or AC-Hly) that enables them to overcome the innate immune defense of the host. CyaA subverts host phagocytic cells by an orchestrated action of its functional domains, where an extremely catalytically active adenylyl cyclase enzyme is delivered into phagocyte cytosol by a pore-forming repeat-in-toxin (RTX) cytolysin moiety. By targeting sentinel cells expressing the complement receptor 3, known as the CD11b/CD18 (α_M_β_2_) integrin, CyaA compromises the bactericidal functions of host phagocytes and supports infection of host airways by *Bordetellae*. Here, we review the state of knowledge on structural and functional aspects of CyaA toxin action, placing particular emphasis on signaling mechanisms by which the toxin-produced 3′,5′-cyclic adenosine monophosphate (cAMP) subverts the physiology of phagocytic cells.

## 1. Introduction

Three species of the Gram-negative aerobic coccobacilli of the genus *Bordetellae*, *B. pertussis*, *B. parapertussis* and *B. bronchiseptica*, are pathogenic to mammals. Of them, the species *B*. *pertussis* is one of the few human-adapted pathogens and causes the respiratory infectious disease called pertussis, or whooping cough [1]. Despite global vaccination coverage, pertussis remains the least-controlled vaccine preventable infectious disease. A recently published model estimates that, in 2014, the global pertussis burden in children younger than five years was ~24 million cases, accounting for more than 160,000 deaths, predominantly of infants [2]. *B. pertussis* infections are often complicated by secondary infections and pneumonia [3], because of a predisposing immunosuppressive action of the numerous virulence factors produced by the pathogen [1]. Among them, the adenylate cyclase toxin–hemolysin (known as CyaA, ACT or AC-Hly) excels in subversion of multiple host immune defense mechanisms [4,5].

**Figure 1:**
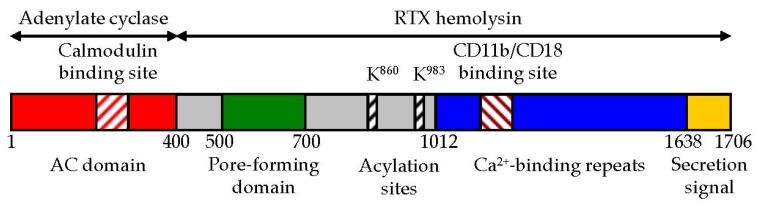
Schematic representation of CyaA. CyaA is a 1706 residue-long polypeptide that consists of an N-terminal AC enzyme domain (~400 residues) and a C-terminal Hly moiety (~1300 residues) that are linked together with a ~100 residue-long segment (residues 400 to 500). The Hly portion of CyaA itself harbors several functional subdomains: (i) a hydrophobic pore-forming domain (residues 500 to 700); (ii) an activation domain (residues 800 and 1000), where the posttranslational acylation at two lysine residues (K860 and K983) occurs; (iii) a typical calcium-binding RTX domain with the nonapeptide repeats binding calcium ions and with the CD11b/CD18-binding segment (residues 1166–1287); and (iv) a C-terminal secretion signal.

CyaA belongs to the large family of repeat-in-toxin (RTX) cytotoxins that harbor the characteristic C-terminal calcium-binding aspartate and glycine-rich nonapeptide repeats of a consensus sequence G-G-X-G-X-D-X-X-X (X, any amino acid residue) [6,7,8]. The toxin is a 1706 residue-long polypeptide (Figure 1) consisting of an N-terminal ~400 residue-long adenylate cyclase (AC) enzyme that is linked to a characteristic RTX hemolysin (Hly) moiety of ~1300 residues [9]. The Hly moiety itself consists of four functional subdomains [7,10], comprising: (i) a hydrophobic pore-forming domain [11]; (ii) an activation domain, with the two posttranslationally acylated lysine residues [12,13]; (iii) a receptor-binding RTX domain consisting of ~40 typical calcium-binding RTX nonapeptide repeats [14]; and (iv) a non-processed C-terminal secretion signal recognized by a bacterial type I secretion system (T1SS) [15,16], respectively.

The Hly moiety of CyaA mediates toxin binding to the CD11b/CD18 heterodimer that serves as the complement receptor 3 (CR3) on myeloid phagocytes, and is also known as the α_M_β_2_ integrin or Mac-1 [17,18]. Upon insertion into the target cell membrane, the Hly moiety delivers the catalytic AC domain of CyaA directly into the cytosol of cells by a poorly understood mechanism that requires proper calcium-induced folding, acylation and structural integrity of the Hly moiety [10,19,20,21]. In the cytosol, the AC domain binds calmodulin (CaM) [22] and hijacks cellular signaling by unregulated conversion of cytosolic adenosine triphosphate (ATP) to the key signaling molecule, 3′,5′-cyclic adenosine monophosphate (cAMP) [4,23,24]. The Hly moiety of CyaA is functionally independent of the AC domain and can itself form small cation-selective membrane pores that permeabilize target cell membrane and can provoke colloid-osmotic (oncotic) lysis of cells [11,19,25,26,27].

Once inserted across the membrane of target cells, CyaA acts as a swift multifunctional saboteur that ablates phagocyte functions by at least three parallel and synergic cytotoxic activities that hijack cellular signaling. Membrane insertion of CyaA mediates: (i) influx of calcium ions into cytosol of cells [28]; (ii) translocation of the AC domain that subverts cellular signaling pathways by an extremely rapid and uncontrolled elevation of cytosolic cAMP concentration [18,23,29,30,31]; and (iii) formation of the oligomeric CyaA pores that permeabilize cellular membrane and activate MAPK signaling by mediating potassium ion efflux from cells [26,32,33,34,35,36]. Depending on the encountered toxin amount, the phagocyte then undergoes apoptotic or necrotic cell death [27,37,38,39].

Here, we summarize the current knowledge of structure–function relationships that underlie the action of CyaA toxin. We next discuss the recent advances in deciphering of the signaling pathways through which the action of CyaA abrogates the sentinel functions of phagocytic cells and enables *Bordetellae* to overcome the innate immune defense of the host.

## 2. CyaA Structure

### 2.1. The AC Domain of CyaA

The AC domain of CyaA belongs to the class II of adenylyl cyclase enzymes [EC 4.6.1.1] that convert ATP to cAMP, a “second messenger” and key intracellular signaling molecule [40]. The AC of CyaA of *Bordetellae* [5,8], the edema factor (EF) of *Bacillus anthracis* [41,42] and the ExoY effector of *Pseudomonas aeruginosa* [43,44] play an important role in virulence of these pathogenic bacteria. These AC enzymes exhibit a very low catalytic activity inside bacterial cytosol and are only activated upon delivery into cytosol of host eukaryotic cells, where ExoY is activated by filamentous actin [45], while the EF and the AC domain of CyaA are activated upon binding of eukaryotic CaM in a 1:1 stoichiometry [22,41,46]. Binding to CaM increases the catalytic activity of the AC enzyme by a factor of ~100–1000-fold [22,47] and the CaM-activated AC enzyme produces a pathologic level of cytosolic cAMP that hijacks cellular signaling [4,24]. CyaA catalyzes not only the formation of cAMP but also the formation of cCMP and cUMP and these multiple cNMP-forming enzyme activities involve a single catalytic site [48]. Two functional subdomains of the catalytic AC domain, T25 and T18, were identified by limited proteolysis, which are still able to assemble with CaM into a fully active ternary complex [46]. By using residue modification and mutagenesis, the catalytic site of the AC was located within the T25 subdomain (residues 1–224), while the main CaM-binding site was located on the T18 fragment (residues 225–399) [49,50].

**Figure 2:**
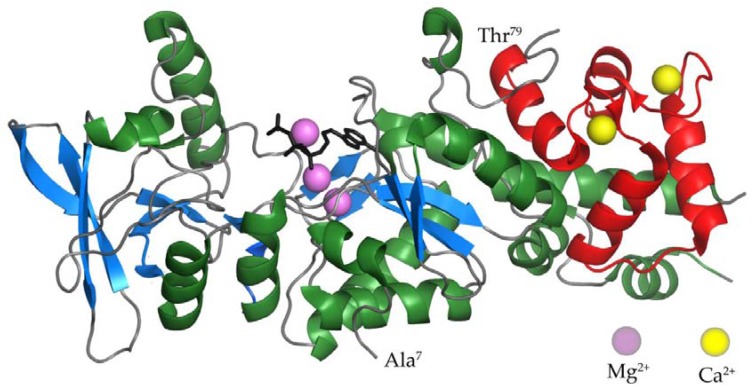
The crystal structure of the AC domain of CyaA in the complex with the C-terminal domain of CaM [53]. α-helices and β-strands of the AC domain are colored in green and blue, respectively. The C-terminal domain of CaM is colored in red. Calcium and magnesium ions are represented by yellow and violet balls. The structure of the ATP analog adefovir diphosphate is represented by black lines.

Several residues play a critical role in the catalytic activity of the AC enzyme of CyaA. Two lysine residues at positions 58 and 65 (K58 and K65) within segment I (residues 54 to 77) were found to be part of the AC catalytic site and their replacements resulted in an important decrease, or total loss, of AC enzymatic activity [49]. In addition, a histidine residue at position 63 (H63) in segment I has been identified as the general acid/base catalyst in a predicted charge-relay system proposed to be involved in the reaction mechanism of adenylyl cyclization [51]. In segment II (residues 184 to 196), two aspartate residues D188 and D190 appear to be required for binding of the Mg^2+^-ATP complex [52]. Finally, substitutions of residues H298 and E301 in segment III (residues 294 to 314) also affect the nucleotide binding properties of AC, albeit to a lesser degree [52]. These early observations were confirmed and corroborated upon solving of the crystal structure of the AC enzyme-C-terminal domain of CaM (C-CaM) complex with adefovir diphosphate (Figure 2), a metabolite of an anti-viral drug tightly binding into the catalytic site and thereby mimicking binding of ATP [53]. This study revealed that D188, D190 and H298 residues are crucial for binding of the catalytic Mg^2+^ metal ions, while D304 is involved in positioning of ribose, and R37, K58, K65 and K84 residues are required for binding of the triphosphate of the ATP substrate [53]. Deprotonation of 3’ OH of ATP is then accomplished by H63, the central catalytic residue of the AC enzyme that is further involved in the reaction mechanism of adenylyl cyclization [51].

The crystal structure of the AC enzyme-C-CaM complex (Figure 2) revealed that four discrete regions of the AC enzyme bind calcium-loaded CaM with a large buried contact surface [53]. Of those, the W242 residue within an α-helix of the T18 subdomain makes extensive contacts with the calcium-induced hydrophobic pocket of CaM [54]. Amino acid substitutions of the W242 residue have yielded AC enzymes with a full catalytic activity and up to 1000-fold reduced affinity for CaM [49,50]. It was further confirmed that all four regions of the AC domain contribute to CaM binding and that the CaM-induced conformational change of CyaA is crucial for catalytic activation [49,50,54]. Biochemical and molecular modeling studies revealed that the β-hairpin (residues 259 to 273) of the AC domain interacts with the N-terminal domain of CaM (N-CaM) [55]. Site-specific mutations in the β-hairpin resulted in conformational perturbations in metal binding sites I and II of N-CaM, while no significant structural modifications were observed in C-CaM [56]. Intriguingly, when the AC domain interacts with intact CaM, the metal-binding sites I and II of CaM bind Mg^2+^ ions, while the sites III and IV of CaM are loaded with Ca^2+^ ions [57]. Mg^2+^ ions are present in millimolar concentrations in the cytosol of cells and the action of CyaA and cAMP signaling induces entry of Ca^2+^ ions into cells [28,58,59]. The competition of Ca^2+^ and Mg^2+^ ions for the metal binding sites I and II offers space for fine-tuning of the catalytic activity of the AC enzyme, which starts to be inhibited already at >100 nM free Ca^2+^ [60].

### 2.2. The AC to Hly-Linking Segment

A ~100-residue-long segment, located between residues 400 and 500 of CyaA (Figure 1), links the AC enzyme to the Hly moiety. It has no homologs in the other proteins of the RTX family and only little is known about its structure and function in CyaA activities. A synthetic peptide comprising residues 454 to 484 of CyaA was shown to possess a lipid bilayer interacting and destabilizing capacity [61]. Mass spectrometry combined with circular dichroism revealed that the linker segment consisting of residues 411 to 490 forms α-helical secondary structures and inserts into the liposomal membrane [62]. In line with the obvious assumption that the AC-to-Hly-linking segment is involved in translocation of the AC domain across the cell membrane, deletion of the residues 375 to 485 abolished the capacity of CyaA to translocate the AC domain across the membrane of erythrocytes [63]. Moreover, this segment is rich in arginine residues proposed to be involved in target membrane destabilization and individual alanine substitutions of four of them (R435A, R443A, R461A and R487A) decreased the capacity of CyaA to translocate the AC domain across target cell membrane [62]. The AC-to-Hly-linking segment further appears to play a role in restricting the propensity of CyaA to form pores in target cell membranes. The CyaA variant lacking the N-terminal residues 6 to 489 exhibits, indeed, a strongly enhanced cell-permeabilizing and pore-forming activity [64,65]. Furthermore, combined substitution of three negatively charged residues clustered within the N-terminal half of the linker segment of CyaA by neutral residues (D445N + D446N + E448Q) provoked a dramatic increase of the specific pore-forming capacity of the toxin without altering its specific capacity to translocate the AC domain across target cell membrane [62]. Thus, the presence and clustering of negative charges in the AC-to-Hly-linking segment of CyaA appear to account for the relatively modest cell-permeabilizing capacity of CyaA pores, as compared to typical hemolysins of the RTX family [62].

### 2.3. The Pore-Forming Domain of CyaA

The hydrophobic pore-forming domain of CyaA, comprised between residues 500 to 700 (Figure 1), consists of several predicted amphipathic and hydrophobic α-helical structures. These play a key role both in AC domain translocation, as well as in the formation of cation-selective oligomeric CyaA pores within target membranes [11,19,25,66,67,68,69]. By using the method of osmotic solute exclusion, the size of the pore formed by CyaA in the cell membrane was estimated to be between 0.6 to 0.8 nm in diameter [26]. Very similar size of CyaA pores was derived also from planar lipid bilayer measurements [11]. The CyaA pores can permeabilize cell membranes for small cations [34] and can provoke colloid-osmotic (oncotic) lysis of erythrocytes, thus accounting for the hemolytic halo that surrounds *Bordetella* colonies grown on blood agar plates [70]. However, compared to typical RTX hemolysins, the specific hemolytic activity of CyaA is rather low [19,26,65,71,72]. This suggests that the pore-forming domain of CyaA evolved towards a function in delivery of the invasive AC domain across target cell membrane, at the expense of its pore-forming capacity. However, the CyaA pore is too small in diameter to allow passage of even an unfolded AC domain polypeptide across the membrane. Moreover, mutant CyaA variants (e.g., E570Q + K860R) that are essentially unable to form pores and permeabilize cellular membrane for monovalent cations, are still fully capable to translocate the AC domain across the lipid bilayer of cell membrane [69] by an as yet enigmatic mechanism, which likely involves formation of a protein–lipid interface by membrane-inserted CyaA monomers. The cation-selective CyaA pores mediate potassium ion efflux from nucleated cells [32,69] and this cell-permeabilizing activity of CyaA contributes to its overall cytotoxicity towards phagocytic cells [27,38,69].

The pore-forming domain can mediate insertion of CyaA into naked lipid bilayers of liposomes on its own [73]. Indeed, truncated CyaA constructs lacking the entire RTX domain and the C-terminal secretion signal (residues 1007 to 1706) can also insert into artificial planar lipid bilayers with applied membrane potential and form pores that exhibit similar characteristics as the pores formed by the entire CyaA toxin [11]. However, the calcium-loaded and folded RTX domain appears to play a key structural role in the pore-forming activity of CyaA as well [7]. The exceedingly cooperative calcium-dependent folding of the RTX domain [7,15,74,75] was found to yield a steep (~50-fold) increase of the propensity of CyaA to form pores in artificial lipid bilayers as the concentration of free calcium ions crosses a very narrow threshold of 0.7 to 0.8 mM Ca^2+^ [76]. CyaA can further insert into and perturb the structure and integrity of lipid bilayer membranes that are devoid of membrane potential and it mediates the release of marker substances from liposomes [20,73,77,78]. However, it is highly questionable whether in the absence of membrane potential the CyaA protein adopts the same transmembrane topology inside lipid bilayer and forms the same types of pores, permeabilizing the lipid bilayer by the same mechanism, as in the presence of membrane potential. This is rather unlikely, as the phenotypes of charge-reversing substitutions of the E509 and E516 residues within the pore-forming domain of CyaA, which significantly alter the hemolytic and pore-forming activities of CyaA on membranes bearing potential [68], are not at all reproduced on liposomal membranes that are devoid of membrane potential [73]. Moreover, CyaA pores were shown to exhibit voltage dependence and the type and characteristics of pores formed by CyaA were found to depend on the level and orientation of the electrical potential across the membranes [79]. Therefore, interpretation of results obtained with CyaA on liposomal membranes is highly prone to generate artifactual and biologically irrelevant conclusions.

### 2.4. The Activation Domain of CyaA

Production of the biologically active CyaA toxin requires covalent posttranslational fatty-acyl modification of proCyaA by the co-expressed protein toxin acyltransferase CyaC [80,81]. Initially, the native CyaA purified from *B. pertussis* (BP338) was found to be activated by amide-linked palmitoylation of only the ε-amino group of the lysine residue 983 [12]. However, the recombinant CyaA produced in *E. coli* in the presence of CyaC was found to be acylated also on the lysine residue 860 [13]. A double modification of both conserved lysine residues (K860 and K983) was then found also on CyaA secreted by the recombinant *B. pertussis* strain 18323/pHSP9, which produces increased CyaC and CyaA amounts due to expression of the *cyaCABDE* locus from a low copy number plasmid [82]. It remains to be determined whether CyaA produced by clinical isolates is doubly acylated or mono-acylated.

Ablation of either of the acylation sites by an arginine substitution yielded monoacylated CyaA-K860R and CyaA-K983R toxins that exhibited importantly reduced toxin activities on erythrocytes that lack the CD11b/CD18 integrin receptor of CyaA [83,84]. However, subsequent production of CyaA with both K860 and K983 residues intact, but preferentially acylated on either the K860 or the K983 residue by co-expressed mutated variants of CyaC, allowed concluding that the K860 residue plays a structural role in toxin activity that is independent of its acylation status [85]. Use of such incompletely acylated CyaA variants allowed to demonstrate that acylation of the K860 residue alone does not confer a substantial cell-invasive activity on CyaA. In contrast, acylation of K983 was found to be necessary and sufficient for toxin activities on erythrocytes [85]. This was confirmed also for toxin activities on myeloid phagocytic cells that bear the CD11b/CD18 receptor of CyaA [84]. Acylation of the K860 residue alone conferred on the monoacylated CyaA-K983R toxin an almost full capacity to bind the receptor and tightly associate with the CD11b/CD18-expressing J774A.1 mouse macrophage cells but the capacity of CyaA-K983R toxin to penetrate cells and translocate the AC domain was largely impaired [84]. In contrast, the monoacylated CyaA-K860R was as active in CD11b/CD18 binding and AC domain delivery into the macrophage cells as the intact CyaA, demonstrating that the acylation of K860 is dispensable, whereas the acylation of the K983 residue is necessary and sufficient for the capacity of CyaA to bind and penetrate the membrane of myeloid phagocytes [84].

Of note, the non-acylated pro-CyaA forms pores in planar lipid bilayers with a lower propensity than the fully acylated toxin, but the proCyaA pores have similar properties as pores formed by the acylated CyaA [84]. It appears, hence, that the presence of acyl residues on CyaA is not crucial for the structure of the formed pores. However, pores formed in lipid bilayers by the monoacylated CyaA-K983R toxin exhibited importantly reduced selectiveness for cations than pores formed by intact CyaA, revealing that the K983 residue may also play an important role in toxin structure independently of its acylation status [84].

### 2.5. The RTX Domain of CyaA

**Figure 3:**
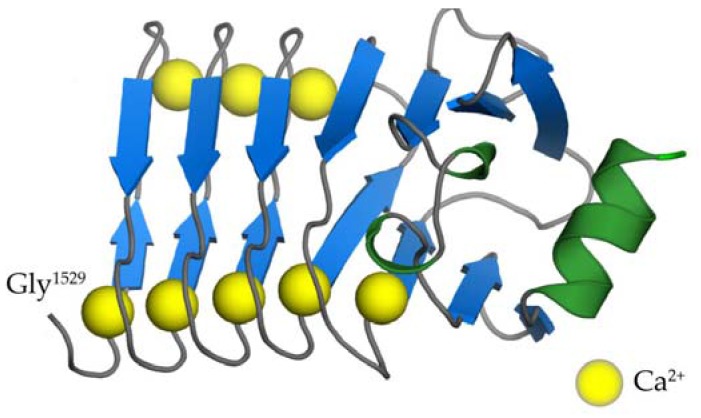
The crystal structure of the segment 1529–1681 of CyaA [15]. The consecutive nonapeptide tandem repeats (G-G-X-G-X-D-X-X-X) are arranged in a regular right-handed helix of parallel β-strands (β-roll). β-strands and the C-terminal α-helix are colored in blue and green, respectively. Calcium ions are represented by yellow balls.

The RTX domain of CyaA consists of five distinct RTX blocks (I to V) located between residues 1012 and 1638 that harbor the typical RTX nonapeptide repeats [14]. Recently, we solved the 3D structure of the segment encompassing residues 1529 to 1681 of CyaA and comprising the block V with its C-terminal folding-initiating structure (Figure 3) [15]. The nonapeptide tandem repeats within this structure are arranged in a regular right-handed helix consisting of parallel β-strands, forming a β-roll structure with eight calcium ions bound within the turns connecting the β-strands [15]. Similar β-roll arrangement was observed for 3D structures of other RTX proteins, such as proteases and lipases of *Pseudomonas aeruginosa* and *Serratia marcescens* [86,87,88]. In these typical RTX β-rolls, the first six residues of the nonapeptide RTX motif (G-G-X-G-X-D) constitute a turn with a bound calcium ion, while the last three residues form a short β-strand. Calcium ions are then periodically coordinated by carboxyl groups of the side chains of aspartic acid residues and the carbonyl groups of the polypeptide backbone of the nonapeptide RTX motifs.

The five β-roll blocks of the RTX domain are connected by segments of variable lengths and their Ca^2+^-dependent folding proceeds in an extremely cooperative manner and results in dehydration and compaction of the RTX domain [7,15,74,75,79]. As recently revealed by a low-resolution SAXS structure of the entire monomeric CyaA holotoxin molecule, the N-terminal ~1000 residue-long portion of the CyaA molecule then can fold back onto this RTX domain “scaffold” to form a rather compact and quite stable structure [89]. Finally, the blocks II and III of the RTX domain form the major CD11b/CD18-binding structure of CyaA [18,90,91].

### 2.6. The C-Terminal Secretion Signal of CyaA

The Hly moiety of CyaA harbors within the last 74 residues an unprocessed secretion signal that is recognized by the T1SS apparatus formed by the CyaBDE proteins across the bacterial cell wall [16,92]. Led by the C-terminal sequence, the toxin is extruded through the T1SS conduit in an unfolded state, directly from the calcium-depleted bacterial cytosol into the host body fluids containing millimolar concentrations of free calcium ions [9,15,93,94]. At the low concentrations of calcium ions inside the bacterial cytosol, the RTX domain remains intrinsically disordered and highly hydrated [75,95,96,97]. In the course of extrusion from bacterial cells, the binding of calcium ions from body fluids triggers folding of the emerging C-terminal segment [98]. This forms a capping structure and scaffolds the calcium-driven co-secretional folding of the translocating RTX domain, which proceeds vectorially from the C-terminus towards the N-terminal end and ratchets and accelerates toxin translocation out of the bacterial cell [15].

## 3. Binding Interaction of CyaA with the Complement Receptor 3 on the Surface of Phagocytic Cells

Due to the extremely high specific enzymatic activity of the AC domain of CyaA, the toxin was found early on to bind and penetrate at low but well detectable levels a variety of eukaryotic cell types originating from diverse mammalian species [24,71,99,100]. This non-saturable and unspecific cell binding is likely to be mediated by a low affinity interaction of the toxin with abundant cell surface glycoproteins, or glycosylated lipid molecules, such as certain gangliosides that accumulate in lipid microdomains of the plasma membrane of cells [20,101]. Indeed, pre-incubation of the toxin with gangliosides, such as >1 μM G_T1b_, strongly reduced the capacity of CyaA to penetrate into Chinese hamster ovary (CHO) cells [20]. However, CyaA was observed to act particularly efficiently, at very low toxin concentrations, on myeloid phagocytic cells, such as neutrophils, monocytes and macrophages [23,102]. This indicated the existence of a proteinaceous and leukocyte-specific receptor of CyaA. Indeed, respiratory infection experiments with *Bordetella* species in mice revealed that CyaA specifically targets neutrophils and other phagocytes without provoking much damage of the airway epithelial cells [103,104]. Moreover, other members of the RTX toxin family were previously found to bind β_2_ integrin receptors of leukocytes [105,106,107,108]. Guermonprez and coworkers demonstrated in 2001 that CyaA specifically binds target cells through interaction with the integrin CD11b/CD18 that serves as complement receptor 3 (CR3) on myeloid phagocytic cells [17].

**Figure 4:**
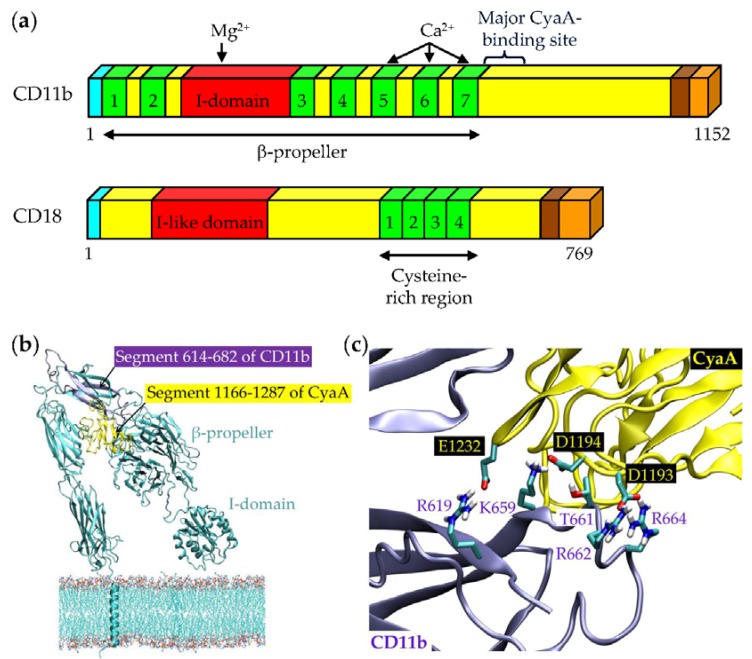
Schematic representation of the CD11b/CD18 heterodimer and its interaction with CyaA. (**a**) The 1152 residues long CD11b subunit contains an N-terminal secretion signal (16 residues; cyan), an N-terminal extracellular domain (1088 residues; yellow), a transmembrane segment (24 residues; brown) and a C-terminal cytoplasmic tail (24 residues; beige) [113,114]. The N-terminal part of CD11b consists of seven β-sheet repeats (1 to 7, from 52 to 79 residues; green), which have been predicted to fold into a β-propeller domain and a region of 187 residues, called the inserted or I-domain, that is localized between repeats 2 and 3 of the β-propeller (red) [115,116]. The I domain contains an Mg^2+^/Mn^2+^ coordination site at its surface (MIDAS) that is critical for ligand binding [115,117,118]. Repeats 5 to 7 of CD11b have EF hand-like Ca^2+^-binding motifs [116]. CyaA primarily recognizes the CD11b segment containing residues 614 to 682. The CD18 subunit is a 769 residues long polypeptide chain harboring an N-terminal secretion signal (22 residues; cyan), an N-terminal extracellular domain (678 residues; yellow), a transmembrane segment (23 residues; brown) and a C-terminal cytoplasmic domain (46 residues; beige) [119]. The extracellular domain contains the I-like domain (residues 124 to 363; red) and 56 cysteine residues forming the Cys-rich region localized near the membrane and composed of four repeat units of approximately 40 residues (green). These fold into small, very compact domains and are parts of the long stalk. The CD11b and CD18 subunits harbor several potential N-glycosylation sites (N-X-S/T), most of them modified by oligosaccharide chains linked to asparagine residues [120,121]. (**b**) 3D model of the CD11b subunit of CR3 (generated with the Modeler suite of programs) with the highlighted segment 614–682 (violet) that interacts with the segment 1166–1287 of CyaA (yellow; modeled using the structure prediction server I-TASSER) [18]. (**c**) The negatively charged residues of the 1166–1287 segment of CyaA are involved in the interaction with the positively charged and hydrophilic residues of the 614–682 segment of CD11b [18]. Reproduced and modified from [18], 2015, eLife Sciences Publications.

The CD11b/CD18 heterodimer (Figure 4a) binds a large variety of physiological ligands and is involved in numerous leukocyte functions. These include adherence of monocytes and neutrophils to vascular endothelium, phagocytosis of complement-opsonized microorganisms, respiratory burst or degranulation of neutrophils, cell spreading, chemotaxis and other functions [109]. CD11b/CD18 belongs to the β_2_ family of integrins restricted to leukocytes, comprising four heterodimeric transmembrane glycoproteins sharing the same β_2_ subunit that pairs with four distinct α subunits [109,110] to form the α_L_β_2_ (CD11a/CD18, LFA-1), α_M_β_2_ (CD11b/CD18, Mac-1, CR3), α_X_β_2_ (CD11c/CD18, p150/195), and α_D_β_2_ (CD11d/CD18) heterodimers, respectively. Despite high level of sequence homology between the α subunits of the four β_2_ integrins, CyaA was found to specifically bind only to dendritic cells (DCs) and macrophages expressing the CD11b/CD18 molecule, but not to B cells or T cells expressing only the CD11a/CD18 molecule [17]. Moreover, cell-binding and penetration of these cells is selectively blocked by certain CD11b-specific antibodies and dose-dependent saturable binding of CyaA is observed only with CHO cells transfected to express CD11b/CD18, but not with CHO cells expressing CD11a/CD18 or CD11c/CD18 integrins [17,18]. At less than 1 nM CyaA concentrations, mimicking the physiological situation in vivo [111], the binding of CyaA to CD11b/CD18 was shown to be a prerequisite for efficient toxin penetration into cells and high levels of intoxication of cells by cAMP, leading to CyaA-induced cell death [27,38,112].

We showed that the initial interaction of CyaA with CD11b/CD18 depends on the recognition of N-linked oligosaccharide chains of the integrin receptor, where a significant reduction of CyaA binding is observed upon removal of the peripheral sialic acid residues from the cell surface glycoproteins by neuraminidase treatment [122]. Moreover, CyaA binding was completely abolished when sugar chains were entirely removed by PNGase F and a complete inhibition of CyaA binding to the CD11b/CD18-expressing cells was observed when N-glycosylation of de novo synthesized proteins was blocked by tunicamycin, while the CD11b/CD18 heterodimer still formed and was presented on cell surface [122]. In addition, CyaA binding to the integrin receptor was specifically and efficiently inhibited in the presence of free saccharides that are found as building units of the oligosaccharide chains of CD11b/CD18 [122].

The CD11b subunit harbors 19 predicted canonical N-glycosylation sites (N-X-S/T) and the CD18 subunit has 6 such sites [109]. To analyze the relative importance of individual N-glycans of CD11b/CD18 for binding and biological activity of CyaA, we substituted one-by-one the asparagine residues of each of the consensus N-glycosylation sites of CD11b/CD18 with a glutamine residue that cannot be glycosylated [120]. Examination of the CD11b/CD18 mutant variants and mass spectrometry analysis of the N-glycosylation pattern of the integrin revealed that N-linked oligosaccharide chains located in the C-terminal portion of the CD11b subunit are involved in CyaA binding and penetration into cells [120]. The initial interaction of CyaA with CD11b/CD18, hence, appears to depend on a multivalent interaction of CyaA with multiple oligosaccharide chains located in the C-terminal portion of the CD11b subunit. These multivalent contacts with glycan chains then would enhance both the specificity and the affinity of the initial interaction of CyaA with its receptor.

The weak lectin-like capacity of CyaA to bind sugar structures offers an attractive hypothesis for explaining the previous observation that CyaA may bind gangliosides on cells lacking the CD11b/CD18 receptor [20,101]. Gangliosides are acidic lipids composed of a ceramide linked to an oligosaccharide chain containing one or more sialic acid residues [123]. CyaA might thus potentially recognize an oligosaccharide moiety of gangliosides, including negatively charged sialic acid residues, similarly as it recognizes oligosaccharide chains of the β_2_ integrin CD11b/CD18.

Upon a weak primary interaction of CyaA with the N-linked glycans, the selectivity of CyaA binding to CD11b/CD18 is dictated by a subsequent highly specific protein–protein interaction. It involves the segment 1166–1287 of CyaA [90] and a segment containing residues 614 to 682 of the CD11b subunit of CR3 (Figure 4b) [18]. Detailed analysis of the binding interaction revealed that negatively charged glutamate and aspartate residues of the 1166–1287 segment of CyaA would interact with positively charged and hydrophilic residues within the 614–682 segment of CD11b (Figure 4c) [18]. However, the interaction may be more complex, as additional segments of CD11b appear to be contributing to CyaA binding to the CD11b/CD18 heterodimer, to some extent [18]. This suggests that the toxin binds the integrin through a multivalent interaction also at the protein–protein level. Engagement of other CD11b segments may facilitate or stabilize/position the toxin for higher-affinity interaction with the principal binding site located within the 614–682 segment of CD11b. Moreover, binding of CyaA to CD11b required proper folding of the CD11b subunit on the scaffold of the CD18 subunit, as CyaA bound CD11b only when this was associated and presented in a complex with CD18 on cell surface [18]. Most importantly, unlike fibrinogen, iC3b, the intercellular adhesion molecule 1 (ICAM-1) and other proteinaceous ligands of CR3, the CyaA toxin did not bind the integrin through the conserved I (inserted)-domain of CD11b (Figure 4) [18,115].

These data allowed us to propose that the segment between residues 614 to 682 and the adjacent N-linked oligosaccharide chains located in the C-terminal portion of CD11b may cooperate in forming a highly organized structure that accounts for specific binding of CyaA to CD11b, supporting its subsequent penetration across cellular membrane and biological activities. The structural integrity of this CyaA-binding site could be destabilized by deglycosylation of CD11b [122], by the removal of some of the individual N-linked glycans [120], or by point substitutions introduced into the 614–682 segment [18]. We further demonstrated that the transmembrane α-helical segments of CD11b/CD18 are not directly involved in AC domain translocation and/or pore formation by the toxin but serve in maintaining of a proper conformation of the integrin, required for efficient toxin binding and action [124].

## 4. Phagocyte Membrane Penetration and Permeabilization by CyaA

**Figure 5:**
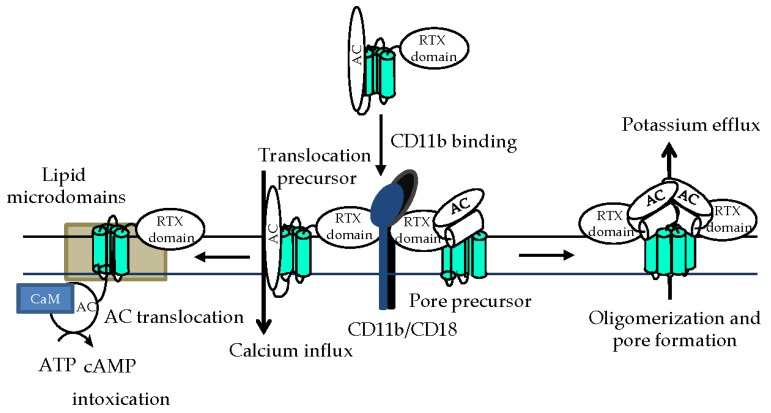
Schematic model of CyaA action on target membrane. After binding to CD11b/CD18 on myeloid cells [17,18], CyaA penetrates the cytoplasmic membrane and employs two distinct conformers to exert its multiple activities [68]. One would be the translocation precursor that would account for delivery of the invasive AC domain across the lipid bilayer [69] and provoke a concomitant influx of calcium ions into cell cytosol [28]. The other conformer would form a pore precursor that would oligomerize into CyaA pores [11,66,68], provoking potassium efflux from target cells [34]. Not drawn to scale.

The highly specific interaction with the CD11b subunit of CD11b/CD18 facilitates insertion of CyaA into the lipid bilayer of cellular membrane and translocation of the AC domain across it into the cytosol of phagocytic cells (Figure 5). The irreversible insertion of the toxin into cell membrane shifts the toxin–receptor binding equilibrium to the right and would account for the very high apparent affinity (in the nanomolar range) of CyaA binding to CD11b/CD18-expressing cells [17,18]. Indeed, an about two orders of magnitude lower affinity is observed for the mere binding interaction of CyaA with the isolated CD11b/CD18 heterodimer that is not anchored in a biological membrane [17,18].

The AC domain of the toxin crosses the cellular membrane in two steps (Figure 5). First, the initial insertion of the AC domain into the membrane with the rest of the CyaA molecule creates a transient conduit for calcium and permeabilizes cells for influx of extracellular calcium ions [28]. This activates calpain-mediated cleavage of the talin tether that anchors CD11b/CD18 to actin cytoskeleton and mobilizes the CyaA-integrin complex for recruitment into cholesterol-rich lipid microdomains. There, the specific packing of membrane lipids allows completion of the second step of AC domain translocation across membrane into cell cytosol [58]. AC translocation across the lipid bilayer is driven and controlled by the gradient of electrical potential across the membrane [59,125] and exhibits a very short half-time of dozens of seconds [71].

In parallel to translocation of the AC domain, the hydrophobic pore-forming domain of CyaA is critical for the second activity of the toxin in target membranes (Figure 5), namely for its ability to form oligomeric cation-selective transmembrane pores [9,11,66,68,69,126]. While translocation of the AC domain into cell cytosol appears to be a linear function of toxin concentration, the pore-forming activity of CyaA is a higher order function of toxin concentration and exhibits a Hill cooperativity number higher than 3 [25,34,68,127,128]. This suggests that monomers of CyaA can deliver the AC domain across membrane, bypassing the CyaA pore [69], while oligomerization of several toxin molecules is required for formation of cation-conducting pores. Indeed, pores formed by CyaA in artificial membranes behave as frequently opening and closing membrane channels that exhibit a short lifetime, suggestive of association-dissociation equilibrium between the non-conducting CyaA monomers and the conducting CyaA oligomers [11,66]. Moreover, pairs of inactive CyaA constructs with non-overlapping deletions complemented each other in vitro and exhibited a partially restored cytotoxic activity [10]. Active CyaA complexes were then obtained through association of an inactive CyaA variant, lacking a conserved sequence located between residues 1636 and 1650 (block A), with the C-terminal CyaA fragment (residues 1490 to 1706) carrying the block A [129]. The ability of CyaA to form the oligomeric membrane pores appears to be selectively modulated by the acylation status of the toxin, as only the pore-forming (hemolytic) activity of CyaA, but not its membrane penetration and AC delivery capacities, appear to be differentially affected upon covalent modification of the K860 and K983 residues by different fatty-acyl chains [11,13,83,127]. The rate of formation, as well as the cation selectiveness and size of the CyaA pores can further be altered by charge-reversing substitutions of glutamate residues in the predicted amphipathic transmembrane α-helical segments of the pore-forming domain of CyaA [66,68,130].

Importantly, the influx of extracellular calcium ions into cell cytosol, provoked by toxin insertion into cell membrane, decelerates recycling of cellular membrane and the uptake of membrane-inserted CyaA pores [33]. Persistence of CyaA pores within the cytoplasmic membrane exacerbates pore-mediated efflux of potassium ions from cells, which further decelerates clathrin-dependent endocytic uptake of the membrane-inserted CyaA pores [33]. Cell permeabilization, hence, exacerbates in a positive feedback loop, where enhanced cell permeabilization further decreases toxin pore removal from the membrane and enables the escape of membrane-inserted toxin pores from proteolytic destruction in the endosomes [33].

## 5. Subversion of Spleen Tyrosine Kinase (Syk)-Mediated Signaling by CyaA-Produced cAMP

Like other integrins, also the CyaA receptor CD11b/CD18 is involved in bi-directional signaling across cellular membrane [131,132]. Upon activation by intracellular signals, the inside-out signaling is triggered through rearrangement of the integrin molecule from an inactive (closed, bent, low-affinity) conformation to an active (open, extended, high-affinity) conformation, which enhances the avidity of the integrin for ligands [133,134]. While the endogenous ligands preferentially bind the I-domain of activated CD11b/CD18 [135], CyaA preferentially binds the inactive integrin that is in the bent conformation [18]. By difference to endogenous ligands binding to the I-domain, the I-domain-independent mode of CyaA binding does not trigger outside-in signaling through the canonical Src-ITAM-Syk signaling pathway in human monocytes [18,136,137,138,139,140]. Moreover, the intracellular signaling of cAMP produced by the translocated AC enzyme of CyaA prevents by an as yet unknown mechanism the activation of Syk by the outside-in signaling, resulting from binding of the complement activation product iC3b to CD11b/CD18. Furthermore, CyaA/cAMP signaling can near-instantly inactivate also Syk that has been pre-activated by iC3b binding to CD11b/CD18 [18]. This unprecedented mode of binding and action distinguishes CyaA from all other known ligands of the I-domain-containing integrins and it has numerous implications for our understanding of the potent immunosubversive capacity of CyaA on myeloid phagocytes. Indeed, Syk signaling plays a key role in triggering both FcR- and CR3-mediated phagocytic pathways upon immunoglobulin or iC3b binding to opsonin receptors [141,142,143]. Besides inactivation of Syk, CyaA/cAMP signaling was found to rapidly and selectively decrease also the activity of the small GTP-ase RhoA [31], which acts downstream of Syk [144]. The inhibitory action of CyaA at the two different signaling levels (Syk and RhoA) would thus synergize in bringing about a rapid and complete inhibition of opsonophagocytic uptake and killing of bacteria [23,31,145]. Besides FcR- and CR3-mediated phagocytosis, the Syk tyrosine kinase also regulates other cellular processes involved in the control of bacterial infections by the sentinel cells of the immune system, such as phagocyte chemotaxis, neutrophil extracellular trap (NET) formation, generation of reactive oxygen intermediates and degranulation of phagocytes [137,146,147,148,149]. All these phagocyte activities were previously found to be suppressed by the cAMP-elevating activity of CyaA [23,102,150,151]. The observed disruption of Syk signaling by CyaA/cAMP, hence, impairs a broad range of host innate immune defense mechanisms that are critical for control of *Bordetella* infection.

## 6. Subversion of Airway Sentinel Cell Functions by CyaA

### 6.1. Alveolar Macrophages

Alveolar macrophages (AMs) serve as major sentinel and antigen-presenting cells of the lower airways and their phenotype resembles in many respects that of DCs. In the steady state, the AMs express low levels of the CyaA receptor CD11b/CD18 and upregulate its expression to higher levels upon pro-inflammatory activation in response to infection [152]. While nothing is known about the outcome of CyaA/cAMP intoxication of AMs, this is likely to play a role in modulation of host immunity at least in the course of the critical pneumonia phase of pertussis in infants. At this stage, *B. pertussis* bacteria are massively present in the alveoli, the AMs are activated and CyaA can target them through binding to CR3. It is not known whether *B. pertussis* bacteria reach lung alveoli in any significant numbers, or whether CyaA action on AMs plays any important role in modulation of host immunity, in the course of a typical uncomplicated whooping cough illness in older children or adults. This could occur for example during the catarrhal phase of the disease, when bacteria proliferate vigorously on the mucosa of the upper airways. Moreover, even quiescent AMs express increased levels of pro-inflammatory genes, being ready to initiate a prompt innate immune response [153]. Hence, a potential immunomodulatory action of CyaA on AMs might be involved in dampening of host immune response to *B. pertussis* infection, the hallmark of which is the absence of fever and of any massive pro-inflammatory host response. It is thus plausible to speculate that CyaA subverts the immune signaling initiated by AMs in response to infection and restricts the release of soluble mediators, such as interferons, IL-1β, IL-8, TNF-α, and CXCL5, which attract and activate neutrophils and stimulate their proliferation in bone marrow [154,155]. AM-produced signaling also leads to activation of intraepithelial DCs (IAE-DCs) interspersed between columnar epithelial cells of the airway mucosa, which are another likely target of subversive action of CyaA.

### 6.2. Modulation of Macrophage Functions by CyaA/cAMP Signaling

As introduced above, it remains unknown whether and how does the CyaA toxin subvert the immune functions of AMs. However, there is little doubt on the relevance of CyaA toxin action on monocytes and lung interstitial macrophages attracted to *B. pertussis-*infected airway mucosa [156]. Confer and Eaton in 1982 and Pearson and co-workers in 1987 have shown that CyaA action on primary human monocytes subverts their capacity to respond by reactive oxygen species (ROS) production to diverse stimuli [23,102]. We showed in 2008 that the CyaA-produced cAMP signaling instantly inhibits CR3-mediated opsonophagocytic uptake of particles into mouse macrophages exposed to as little as <0.1 nM purified CyaA toxin [31]. The FcγR-mediated phagocytic uptake of antibody-opsonized particles was less sensitive to interference of CyaA/cAMP signaling. However, CyaA action rapidly inhibited macropinocytic fluid phase uptake and provoked massive actin cytoskeleton rearrangements and unproductive membrane ruffling, due to selective inactivation of the small GTPase RhoA in the absence of any detectable activation of Rac1, Rac2 or RhoG [31].

CyaA activity was found to extend survival of non-opsonized *B. pertussis* bacteria within human macrophages under conditions of high multiplicity of infection [157,158]. Using murine RAW264.7 macrophages, we found that CyaA/cAMP signaling through the protein kinase A (PKA) pathway activates by an as yet unknown mechanism the Src homology domain 2 containing protein tyrosine phosphatase (SHP) 1 that plays an important role in regulation of numerous receptor signaling processes in leukocytes [29]. PKA signaling and activation of SHP-1, but not of SHP-2, inactivated the AP-1 transcription factor through dephosphorylation of its c-Fos subunit and inhibited LPS-inducible expression of the NO synthase (iNOS) and production of bactericidal NO. As a result, the CyaA/cAMP signaling-induced activation of SHP-1 extended the survival of internalized *B. pertussis* bacteria inside murine macrophages [29]. Moreover, CyaA action provoked activation and nuclear translocation of the NF-κB transcription factor, while decreasing the phosphorylation of STAT1 and the protein levels of the IRF1 transcription factor [29]. This is likely to modulate the transcriptional program in macrophages towards immunomodulatory cytokine signaling and M2 polarization.

### 6.3. CyaA/cAMP-Triggered Reprogramming of Gene Expression in Macrophages

Recently, the analysis of global gene expression profiles of CyaA-treated murine bone marrow-derived macrophages (BMDMs) exposed to 100 pM CyaA for 24 h revealed that sustained CyaA-cAMP signaling altered expression of more than 2000 genes. Most of the upregulated ones were coding for proteins involved in immune responses and inflammation, such as genes for DC markers or for chemokines involved in chemoattraction of neutrophils (*Cxcl5* and *Cxcl7*) [159]. Such CyaA activity effects on lung AMs would go well with the observation that a synergy between cAMP signaling and pore-forming activities of CyaA accounts for massive neutrophil infiltration into *B. pertussis*-infected mouse lungs [156].

Among the down-regulated genes were the ones for proteins involved in cell proliferation, such as cyclins B1 and B2 and their CDK1 partner [159]. This would go well with the observation that a single in vitro exposure of murine J774A.1 macrophage cells to non-lethal (picomolar) concentrations of CyaA provokes cell cycle arrest in the G_1_/G_0_ phase, which lasts for 3 to 6 days. This appears to be due to inhibition of phosphorylation of the ERK 1/2 kinase and of cyclin D1, with concomitant increase of levels of the CDK inhibitor p27kip1 and of phospho-CREB [160].

Deeper analysis of gene expression alterations induced in BMDMs by exposure to 100 pM CyaA revealed modulation of several pathways that regulate systemic and local inflammatory responses and chromatin remodeling, such as Smarcc1 (or SRG3). This chromatin remodeling factor was recently implicated in promoting of a Th2 response by reprogramming of DCs or macrophages [161] and in the shift from classical “proinflammatory” (M1) to alternative “immunoregulatory” (M2) activation of macrophages [162]. The decision between the “classical” M1 and “alternative” M2 activation states of macrophages is driven by various mechanisms, of which one includes modulation of subcellular localization and activities of the SIK family of kinases. In line with that, we have recently observed by a phosphoproteomic analysis that CyaA/cAMP signaling triggers posttranslational regulation of SIK kinases and eventually also of their downstream target CRTC3 in murine bone marrow-derived dendritic cells (BMDCs) [163]. This is consistent with the known outcomes of CyaA action on DCs, as SIK1 and SIK3 were shown to negatively regulate the response to TLR4 stimulation [164]. Inhibition of SIK kinases, followed by CRTC3 dephosphorylation, was then shown to promote production of the anti-inflammatory cytokine IL-10, which drives tolerogenic polarization of M2 macrophages [165,166]. Further analysis of the impact on gene expression profiles of BMDMs [159] indicates that CyaA/cAMP signaling triggers atypical activation of macrophages that includes markers characteristic for both M1 and M2 activation states. Experimental testing of this hypothesis is currently underway in our laboratory.

### 6.4. Induction of Macrophage Apoptosis by CyaA

In addition to blocking bactericidal functions of phagocytes, CyaA is also able to induce macrophage apoptosis both in vivo and in vitro [103,167]. First apoptotic cells can be observed after 2 h of incubation of macrophages with the *B. pertussis* 18323 bacteria at multiplicity of infection ratio of 100:1, which in 6 to 8 h yields apoptotic death of almost 100% of infected macrophages [167]. Triggering of apoptotic cell death does not depend on engulfment of *B. pertussis* bacteria [167] and depends on toxin activity of CyaA, but not of pertussis toxin [103,167]. We have recently demonstrated that cAMP-induced PKA signaling results in stabilization of the pro-apoptotic Bcl-2 family protein BimEL [112], most likely through direct phosphorylation of BimEL by PKA at the serine 83 residue [168]. In parallel, PKA by an unknown mechanism causes activation of the SHP-1 tyrosine phosphatase [29] that can dephosphorylate ERK1/2 [169] and thus inhibit its capacity to phosphorylate BimEL and commit it for degradation by the proteasome [170]. Accumulation of BimEL then leads to activation of the Bax protein and its permeabilizing association with outer mitochondrial membrane and release of cytochrome c [112]. In parallel, CyaA-produced cAMP signaling provokes dephosphorylation of threonine 308 and serine 473 residues of the pro-survival kinase PKB/AKT and inhibition of its activity. As a result, the FoxO3a transcription factor becomes dephosphorylated and eventually translocates into cell nucleus, where it up-regulates transcription of the *Bim* gene, yielding a persistent increase of pro-apoptotic BimEL levels in cell cytosol [112].

### 6.5. Intraepithelial Dendritic Cells

IAE-DCs of myeloid origin are found in both the epithelium and submucosa of conducting airways, as well as on the alveolar surfaces [171,172]. During the response to infection, these bona fide DCs are further replenished by monocyte-derived DCs (mo-DCs) [173] and upon activation by TLR ligands, the IAE-DCs and mo-DCs mature and migrate to the lung-draining lymph nodes to trigger the T cell response.

To get a grasp of the complexity of the subversive CyaA/cAMP signaling in host DCs, we have performed a global and unbiased phosphoproteomic analysis of the early phases of cAMP intoxication of mouse BMDCs by CyaA [163]. Among others, this complex approach revealed a cAMP signaling-dependent inhibition of mTOR and of the SIK family kinases-dependent signaling pathways. cAMP-dependent activation of mTOR inhibitors like tuberin [174] or PRAS40 [175] yielded, among others, a hypophosphorylation of 4E-BP1, which likely causes repression of cap-dependent protein translation [176]. Moreover, mTOR is an important regulator of immune responses and its inhibition has numerous pleiotropic effects [177,178,179]. Among others, mTOR inhibition would impact on a number of key processes of DC biology, including maturation, migration or cytokine production [179]. Hijacking of these processes by CyaA/cAMP signaling then likely enables *Bordetellae* to favorably shape the host immune response towards immune evasion. Another highly relevant effect of CyaA/cAMP signaling in BMDCs appears to be the cAMP-dependent manipulation of the phosphorylation state of the SIK family of kinases and dephosphorylation of their downstream target CRTC3/TORC3 [163]. This was previously shown to enhance CREB-dependent transcription of the gene for the anti-inflammatory IL-10 cytokine [165]. In this model, the cAMP-activated PKA (and/or eventually other kinases) would phosphorylate SIK1-3 kinases in a cAMP-dependent manner and inhibit their capacity to phosphorylate CRTC3. Dephosphorylated CRTC3 would be next liberated from the complex with the 14-3-3 protein, would translocate into cell nucleus and would bind CREB, which is itself activated via a CyaA/cAMP-activated cAMP-PKA-dependent pathway [180]. CRTC3 would then act as a transcriptional coactivator of phospho-CREB, promoting IL-10 production in phagocytes and M1 to M2b (regulatory) phenotype shift in macrophages [165].

### 6.6. CyaA Action on Neutrophils

Infection-elicited activation of cytokine and chemokine secretion by TLR-activated IAE-DCs, AMs and infected epithelial cells of the airway mucosa provokes chemotaxis of neutrophils patrolling in the pulmonary vasculature. Neutrophils are the first sentinel cells that enter the infected airways [181,182] and are the most plausible target of the subversive CyaA toxin action, fooling the release of antimicrobial peptides and hydrolytic enzymes from granules of neutrophils and inhibiting assembly of the NADPH oxidase complex and ROS release by oxidative burst of neutrophils [183]. Subversion of the bactericidal activities of neutrophils would thus appear as the primary role of the CyaA toxin in subversion of host immune defense in the early stages of airway infection by *Bordetellae* [23,104,183]. Indeed, even before the exact nature of the CyaA toxin was characterized, Confer and Eaton discovered that urea extracts of *B. pertussis* bacteria elevate cAMP levels in primary human neutrophils and AMs and thereby paralyze their capacity to produce ROS and kill serum-opsonized bacteria [23]. Friedman and coworkers then described the inhibition of chemotaxis and superoxide production by human neutrophils exposed to CyaA, but failed to observe any changes in their phagocytic capacities by using light microscopy [150]. Inhibition of opsonophagocytosis by CyaA-treated neutrophils was, however, observed by Weingart and Weiss, who showed that upon opsonization with human immune serum a *B. pertussis* mutant lacking CyaA was approximately 10 times more efficiently phagocytosed by human neutrophils than the wild-type bacteria [184,185]. Moreover, addition of purified CyaA to human neutrophils incubated with the mutant bacteria led to impairment of their opsonophagocytic capacity [184]. Furthermore, addition of CyaA-neutralizing monoclonal antibodies, or a human convalescent serum containing such antibodies, restored the phagocytic capacity of human neutrophils exposed to CyaA [184]. More specifically, CyaA action was found to inhibit CR3-mediated phagocytosis by human neutrophils [186], showing that CyaA inhibits a broad spectrum of bactericidal activities of neutrophils.

We have recently addressed the mechanism by which CyaA/cAMP signaling inhibits the oxidative burst of human neutrophils [30]. Two converging mechanisms that interfere with NADPH oxidase activation and ROS production were identified. One appears to involve cAMP/PKA–mediated activation of SHP-1, which limits activation of the ERK and p38 kinases required for assembly of the NADPH oxidase complex. In parallel, activation of the exchange protein directly activated by cAMP (Epac) provokes, by an as yet unknown mechanism, the inhibition of phospholipase C (PLC). By inhibiting PLC-mediated production of the protein kinase C–activating lipid, diacylglycerol, the cAMP/Epac signaling thus blocks the bottleneck step of the converging pathways of oxidative burst triggering [30].

This helps to understand why CyaA plays an important role in the early stages of airway colonization by *B. pertussis*, at least in the mouse model of infection [187,188,189,190,191]. Indeed, no CyaA-deficient clinical isolates of *B. pertussis* have been reported yet, by difference to the massive spread of *B. pertussis* strains defective in production of pertactin, or to occasional isolation of strains deficient in production of filamentous hemagglutinin or pertussis toxin. Further, the 5 kb-large *cyaA* gene exhibits a particularly high degree of sequence conservation across the global population of clinical *B. pertussis* strains [192,193], indicating a high selective pressure for conservation of function of the toxin.

CyaA activity accounts for numerous pathological effects elicited by *B. pertussis* infection in mouse lungs, such as the recruitment of inflammatory leukocytes and the induction of pathological lesions [70,103,156,194]. *B. pertussis* mutants, unable to secrete CyaA, or producing an enzymatically inactive but still hemolytic CyaA-AC^−^ toxoid, were found to be impaired in proliferation in mouse airways [156,187,188,195]. We could recently construct the first non-hemolytic *B. pertussis* mutant that secretes a non-hemolytic CyaA toxin (e.g., CyaA–E570Q + K860R) that is intact for delivery of the AC enzyme into CD11b/CD18-expressing myeloid phagocytes, such as neutrophils, macrophages and DCs [69]. Mouse infection experiments with this mutant then revealed that the cell-permeabilizing (hemolytic) activity of the CyaA toxin is not required for the capacity of *B. pertussis* to proliferate in infected mouse lungs [156]. However, the permeabilization of host cells by CyaA synergizes with the AC enzyme activity of the toxin and importantly contributes to the overall virulence and lethality of *B. pertussis* infection. It is particularly involved in the eliciting of signaling that triggers massive neutrophil infiltration into infected lung tissue and thereby contributes to its subsequent inflammatory damage [156].

Somewhat controversial conclusions were previously reached on whether neutrophils are the main target cells of CyaA during *Bordetella* infections. Harvill and coworkers (1999) showed that CyaA was required for *B. bronchiseptica* infections of SCID-Beige mice lacking the B, T and NK lymphocytes, suggesting that CyaA acts on neutrophils, or on phagocytes of the monocyte/macrophage lineage [104]. Moreover, neutropenic mice were equally susceptible to lethal infection by wild-type or CyaA-deficient strains, suggesting that neutrophil depletion removed the main target cell on which CyaA needs to exert its biological activity and CyaA was, hence, not required for successful infection of neutropenic mice [104]. However, upon infection of neutropenic mice with *B. pertussis* strains, Andreasen and Carbonetti observed a role for CyaA action on neutrophils only in the immune (convalescent or passively immunized by serum) mice but not in the naive mice [104,196]. This indicates that neutrophils are not the only CR3-expressing target on which CyaA has to act in the mouse airway to support a successful infection of this non-natural host of *B. pertussis*. To which extent these results reflect the limitation of the mouse model in reproducing the protective immune mechanisms operating in human airways remains unknown. Nevertheless, these results are consistent with the finding that CyaA-neutralizing antibodies can restore phagocytic uptake of serum-opsonized *B. pertussis* by human neutrophils [184,186]. Indeed, CyaA is able to efficiently abolish the bactericidal functions of neutrophils and besides of blocking oxidative burst, it can also inhibit NET formation and neutrophil apoptosis [151]. The same conclusion was recently reached also for the interaction of *B. parapertussis* with human neutrophils [197].

## 7. Conclusions

**Figure 6:**
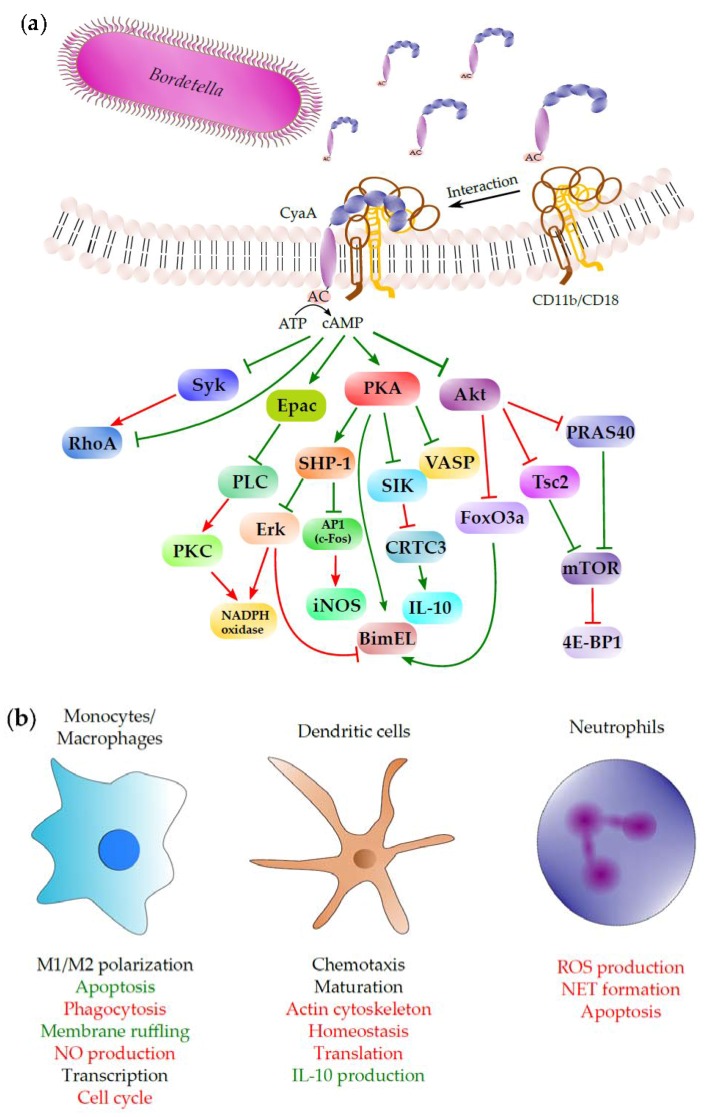
Schematic depiction of CyaA interaction with target cells: cAMP-dependent signaling and its impact on epithelial cells and phagocytes. (**a**) CyaA toxin molecules secreted by the T1SS of *Bordetella* bind specifically to the integrin CD11b/CD18 (CR3, or α_M_β_2_) on the surface of phagocytes (monocytes, macrophages, DCs or neutrophils), or interact nonspecifically with the surfaces of other cell types, including epithelial cells. Following insertion into cytoplasmic membrane, CyaA translocates its enzymatic AC domain into the cytosol. In parallel, membrane-inserted CyaA molecules can oligomerize into cell-permeabilizing pores (not shown). Inside cell cytosol, the AC domain is activated by binding of calmodulin and catalyzes an extremely rapid and unregulated conversion of cytoplasmic ATP into cAMP, a key second messenger that hijacks the different cellular signaling pathways schematically delineated in the drawing (see the text for more detailed description of these pathways). The pointed arrowheads in the drawing indicate an activating effect and the flat arrowheads indicate an inhibitory effect under normal physiological conditions. The red color of the arrowhead indicates an inhibitory effect, or interference, elicited by the cAMP signaling of the CyaA toxin and the green color indicates an enhancing effect of CyaA/cAMP-triggered signaling action. (**b**) The outcomes of CyaA/cAMP signaling are shown together with the cell type for which they were originally described. Green color represents stimulation of particular interaction or process by CyaA action, while red color represents inhibition.

The rise of whooping cough incidence in the last two decades is due to several factors. Among them, the rapid waning of protection conferred by the current acellular pertussis (aP) vaccines, and the limited spectrum of antigens in these vaccines, appears as most worrisome [5]. This situation calls for development of a next generation of pertussis vaccines that would not only confer protection against the severe pulmonary disease caused by pertussis toxin in infants, but would also prevent bacterial infection and more-or-less asymptomatic colonization, which accounts for the current massive transmission rates of *B. pertussis* in highly aP-vaccinated populations of older children and adults. The detailed characterization of the structure–function relationships and mechanisms of action of key virulence factors, such as CyaA, offers the potential of their use as protective antigens in new vaccines. As reviewed here and summarized in Figure 6, CyaA activity interferes with a broad range of functions of phagocytes that are the sentinel cells of innate immunity. CyaA-caused paralysis of opsonophagocytic killing of the bacteria appears to play a key role in the early stages of colonization by *B. pertussis*. This makes CyaA toxoid to a leading antigen candidate for inclusion into the next generation of aP vaccines. Induction of CyaA-neutralizing antibodies by future aP vaccines is expected to enable neutrophils and macrophages in the airway of vaccinated subjects to fully deploy their bactericidal opsonophagocytic killing activities. Such maximizing of the protective potential of the opsonizing antibodies against bacterial surface antigens, comprised in the aP vaccine, is then expected to enhance also protection against *B. pertussis* infection and not only against the clinical disease.
